# Overexposure to COVID-19 information amplifies emotional distress: a latent moderated mediation model

**DOI:** 10.1038/s41398-022-02048-z

**Published:** 2022-07-18

**Authors:** Yi Feng, Wen Gu, Fangbai Dong, Dan Dong, Zhihong Qiao

**Affiliations:** 1 Mental Health Center, Central University of Finance and Economics, Beijing, China; 2 Faculty of Psychology, Beijing Normal University, Beijing, China; 3 Mental Health Center, Henan Agriculture University, Henan, China

**Keywords:** Bipolar disorder, Scientific community

## Abstract

An infodemic has accompanied the COVID-19 pandemic. This study explores whether overexposure to COVID-19-related information amplifies emotional distress. A cross-sectional online survey was conducted in China during the outbreak and after the peak of the pandemic (*N* = 1313). A latent moderated mediation model was built to analyze this relationship. COVID-19 information exposure was found to relate positively with emotional distress, and risk perception mediated the association between them. Additionally, psychological resilience moderated the mediating effects of risk perception. However, five factors of resilience differed in their moderating effects. This study offers theoretical and practical implications apropos clinical intervention and public health management.

## Introduction

*“We’re not just fighting an epidemic; we’re fighting an infodemic.” WHO Director-General Dr. Tedros Adhanom Ghebreyesus at the Munich Security Conference, 2020*

The unprecedented global COVID-19 pandemic [1] has been accompanied by a crisis of public knowledge—an infodemic [2]. The urgency of the pandemic and the need for public awareness have flooded media outlets with indistinguishable COVID-19 facts and rumors [3]. Accurate information is necessary to respond appropriately to the pandemic; however, media overexposure can exacerbate health problems and amplify emotional distress [4]. Similar negative psychological effects of repeated media exposure have been identified after the 9/11 terrorist attack, the Boston Marathon bombings, H1N1 flu, and the western African Ebola epidemic [4–6].

Social media provides crucial information about COVID-19, especially under conditions of social isolation. However, overexposure to COVID-19-related information is associated with negative emotions [7–9]. The mechanisms underlying these effects are incompletely understood or limitedly explored in terms of risk perception and psychological resilience, although both factors significantly influence mental health [10, 11]. Investigating the associations and mechanisms between COVID-19 information exposure and emotional distress is necessary to improve public handling of the infodemic.

This study identifies triggering mechanisms concerning the effects of COVID-19 information exposure on emotional distress by focusing on the mediating and moderating effects of risk perception and psychological resilience. This exploration offers theoretical and practical insights. Theoretically, it enriches the knowledge of media exposure, risk perception, and mental health. Practically, it attracts attention of healthcare workers and governments to the infodemic crisis during information communication. It can also form a practical reference for mitigating mental health challenges related to information exposure, risk perception, and psychological resilience.

### COVID-19 information exposure and emotional distress

Legacy media (e.g., print magazines and newspapers, TV, and radio broadcasting) and particularly social media (e.g., Facebook, Instagram, Twitter, YouTube, TikTok, WeChat, Weibo, QQ, and WhatsApp) provide information on COVID-19 during the pandemic [12]. Health professionals and officials use social media to communicate directly with the public, potentially augmenting the impact of information on public health behaviors [13]. However, social media can feature information overload and surges of fake news [12]. In contrast to legacy media, which filters and supervises information and sources, social media is characterized by interactivity and sharing of information [14], thereby leading to a lack of information control [15]. Access to an overwhelming amount of broadcast material produces an overexposure to information, leads to fear and mental health problems, which harbingers a larger health crisis for societies [4, 16].

Exposure to COVID-19 information is a vulnerability factor for mental health [17, 18], with increased exposure being associated with a higher prevalence of anxiety and depression [8, 9], accounting for approximately 5.1% of the total variance in anxiety [7]. The frequency and duration of COVID-19 information exposure independently predict mental health outcomes [6, 8, 9, 19]. Studies in several countries have consistently found that individuals exposed to COVID-19 information for more than 3 h per day were at a greater risk of developing psychological distress, such as anxiety, depression, and insomnia, than those with less than 1 or 2 h of access [8, 19–21].

China’s netizens now number 989 million, of whom 99.7% use cell phones to obtain information [22]. Global COVID-19 information flows indiscriminately without gatekeepers, through individual mobile devices, and this implies the risk of a Chinese infodemic [23, 24]. Previous studies indicated that increased exposure (in frequency or duration) to COVID-19 information might be associated with increased emotional distress (i.e., anxiety or depression).

### COVID-19 information exposure and risk perception

Risk perception, a crucial concept in health and risk communication, refers to the estimation of the probability of a negative health incident or consequence [25]. It includes perceived susceptibility and severity [26, 27] and encompasses cognitive and affective processes [28, 29]. People tend to immediately perceive risk in a public health crisis [30], especially when confronted with an outbreak of an unexpected infectious disease, including Ebola, MERS, and H1N1 flu [28, 31, 32]. Identifying antecedents and outcomes of risk perception can help the public manage the threats of COVID-19 [33]. This pandemic offers a natural context for studying risk perception in the face of a public health threat.

Mass media is vital in shaping public risk perceptions [34], especially where a health issue is poorly understood by the public. Many people depend on mass media for pandemic information [34]. Media exposure substantially affects public risk perception in many infectious diseases, such as H1N1 flu [28], avian flu [35], and bovine spongiform encephalopathy [26]. However, media exposure exerted various effects on risk perceptions, either attenuating or amplifying [36]. Limited information exposure causes increased uncertainty, uncontrollability, and overestimated risk [5]; overexposure results in likelier exposure to fake or negative news and induces heightened risk perception [4]. Moderate media exposure is crucial: it helps the public connect with reality, utilize resources appropriately, attenuate perceived risk, and comply with preventive policies [37]. Of Chinese citizens, almost 70% are netizens [22], indicating that the majority of Chinese citizens are possibly over-exposed to information. Even though a few people don’t use Internet to obtain information, they can obtain COVID-19 information from channels such as newspapers, radio broadcasting, and TV news. Thus, we propose the assumption solely regarding the case of information overexposure.

Media exposure to information on global outbreaks of infectious disease is positively associated with risk perception and preventive behaviors [33, 39]. Few studies have explored this association in relation to the ongoing COVID-19 pandemic [40]. We assume that COVID-19 information exposure may be positively associated with risk perception in China during the pandemic.

### Risk perception and emotional distress

The perception of danger triggers negative emotions and has adverse mental health consequences, including worry, anxiety, and depression [7, 10, 38]. The WHO specifically indicates that the public overestimates risk vis-à-vis the actual incidence of COVID-19 [1]. Studies in psychology, clinical science, and economics indicate that health-related risks are perceived cognitively and responded to emotionally [41, 42], which generally induces adverse emotional distress [25, 43], having a crucial effect on public mental health in a pandemic [44].

Investigations of the connection between risk perception and mental health have increased during the COVID-19 pandemic [42]. Such studies have consistently reported that COVID-19 risk perception is associated with server emotional distress, such as fear, anger, anxiety, and depression [7, 42, 44–46]. Few studies have investigated the associations between media exposure, risk perception, and emotional distress [47]. Therefore, we assume that risk perception may mediate the influence of COVID-19 information exposure on anxiety and depressive symptoms.

### Resilience and emotional distress

Although exposure to COVID-19 information leads to predictable perceived risk, psychological responses and mental health outcomes vary [48], depending on socio-psychological factors such as personality traits, resilience, and social support [7, 49]. Individuals adopt discrete coping strategies to manage perceived risk. Effective coping supports adaptation to change and maintenance of mental health; however, poor coping results in stress and psychological distress [50]. Coping encompasses a set of adapting skills to adversities, with resilience being a successful outcome [51, 52]. Resilience explains why some people can maintain mental health during crises.

The research on resilience began in child development since the 1970s, and then extended to psychological therapy, disease care, and public health [53]. Resilience refers to the ability to adapt to adversity, trauma, tragedy, threats, or other significant stress [54]. In psychological terms, resilience is considered a defense mechanism [11] and is characterized by two pivotal constructs: adversity and positive adaptation [55]. Current theories consider resilience as a multidimensional concept with stable attributes, such as temperament and personality, along with changeable factors, such as coping and adaptive skills [56, 57]. Thus, resilience offers a short- and long-term positive perspective on mental health, providing due preventive and intervention-related directions for public health crises.

Resilience can support mental health by delivering cognitive, behavioral, and emotional responses in adverse situations [52]. Meta-analyses have identified a positive overall association between resilience and mental health, with a correlation value of 0.48 [52]. Besides, a negative relationship has been found between resilience and psychological distress [57, 58]. Thus, understanding resilience is crucial for developing preventive and intervention strategies to safeguard people from emotional distress in crises [59]. For instance, scholars called for targeted actions after the 9/11 terrorist attacks to enable public recovery [60]. The COVID-19 pandemic is also a stressful public crisis. Presently, resilience is associated with lower COVID-19-related worry, anxiety, and depression [61–63]. It is necessary to examine how resilience continues to influence mental health in this context. A range of studies have defined risk perception as perceived vulnerability to risk [26, 27] and resilience as reduced vulnerability [64, 65]. However, few studies have explored the interactive effects of risk perception and resilience on mental health. This study aims to examine the buffering role of resilience on anxiety and depressive symptoms. More importantly, it aims to investigate how the mediating effects of risk perception on mental health changes with the influence of resilience.

## Method

### Participants and study design

This study applied a cross-sectional design and cluster sampling strategy. It is conducted from February 2 to March 3, 2020, at two Chinese universities in Beijing, where undergraduate and graduate students filled an online questionnaire distributed by their class teachers via a clickable link. During this period, the number of positive cases in China peaked and began to decline and a nationwide self-isolation order was issued (see Fig. S1 in supplemental materials). Each participant could only complete the survey once. The respondents were informed about the purpose of the study and their freedom to withdraw beforehand. Individual informed consent was obtained on the first page of the questionnaire. This study was approved by the Research Ethics Review Committee of Beijing Normal University, China.

With a response rate of 78.6%, 1347 students completed the questionnaire out of the 1713 that participated. The following exclusion criteria were adopted to ensure participation quality [1]: Participants infected by COVID-19 were excluded as most of them were quarantined in isolated wards, and their media exposure was limited [2]. Participants who failed the attention check question: “Please select ‘strongly disagree’ for this question.” [3]. Participants who spent less than 5 min completing the questionnaire as it could not be completed in that time. The time duration was adjusted to 6, 7, or other denominations of minutes. The results of applying different time duration stipulations were all robust. These criteria excluded 34 participants, and the final sample comprised 1313 respondents.

### Measures

#### COVID-19 information exposure

COVID-19 information exposure was assessed through frequency and duration adapted from previous studies [8, 42]. Exposure frequency was measured through a single question: “On average, how many times per day did you browse for COVID-19-related information in the last month?” Responses were rated on a 21-point scale, ranging from “0 times” to “20 times”. Exposure duration was measured by another question: “On average, how many hours per day did you browse for COVID-19-related information in the last month?” Responses ranged on a 13-point scale from “0 h” to “12 h”. Both legacy media and social media were considered. Because of the difference in the scoring method, the correlation of frequency and duration (*r* = 0.33, *p* < 0.001) was calculated to make a reference that the two items had acceptable reliability.

#### Perceived risk

Two items were used to measure perceived risk: “How likely do you think it is that you will be infected with COVID-19” and “How likely do you think it is that your family members/relatives/friends will be infected with COVID-19?” [33, 66]. Participants were asked to rate the probability on an 11-point scale spanning from 0% to 100%. The scores of the two items were totaled to construct a composite score of perceived risk (Cronbach’s α = 0.872); higher scores indicated a greater perceived risk of COVID-19.

#### Emotional distress

Emotional distress encompasses a wide range of emotional suffering typically characterized by symptoms of anxiety and depression [67]. Previous studies proposed that anxiety and depression diagnoses frequently tended to co-occur and their symptoms were highly correlated [68, 69]. Therefore, emotional distress was assessed by two indicators in this study: anxiety and depressive symptoms. Anxiety symptoms were measured using the 7-item Generalized Anxiety Disorder Scale (GAD-7) [70], a self-reporting screening scale that has been validated in China [71]. The participants indicated the occurrence of anxiety symptoms over the past 2 weeks on a 4-point scale (0 = *not at all*; 1 = *several days*; 2 = *more than half the days*; 3 = *nearly every day*). Sample statements included “feeling nervous, anxious, or on edge,” “having trouble relaxing,” and “feeling afraid as if something awful might happen.” A composite anxiety score was calculated by aggregating the scores of all seven items (Cronbach’s α = 0.916); higher scores reflected more severe anxiety symptoms. The cutoff score for the identification of anxiety symptoms was set to 5 [70].

Depressive symptoms were measured using the 9-item Patient Health Questionnaire (PHQ-9) [72]. Similar to GAD-7, this self-reporting screening scale has been validated in China [73]. The participants were asked to indicate the occurrence of depressive symptoms over the past 2 weeks on a 4-point scale ranging from 0 (“*not at all*”) to 3 (“*nearly every day*”). Sample items included “little interest or pleasure in doing things” and “thoughts that you would be better off dead or of hurting yourself in some way.” The scores of the nine items were summed, and a composite index of depressive symptoms was constructed (Cronbach’s α = 0.885). A higher score indicated more severe depressive symptoms. A cutoff score of 5 was used in this study to identify depressive symptoms [72].

#### Psychological resilience

The Connor-Davidson Resilience Scale (CD-RISC) comprising 25 items was used to measure psychological resilience [74]. Participants rated the extent to which they agreed with each statement, examples of which included “I am able to adapt to change” and “I like challenges.” Responses ranged on a 5-point scale (1 = “*not true at all*,” 2 = “*rarely true*,” 3 = “*sometimes true*,” 4 = “*often true*,” 5 = “*true nearly all of the time*”). This scale has been validated among Chinese people [75, 76]. A composite score of psychological resilience (Cronbach’s α = 0.941) was computed by adding all items; higher scores demonstrated greater resilience in handling adversities. Besides, this scale incorporates five factors: tenacity (i.e., personal competence, high standards, and tenacity), tolerance (i.e., trust in one’s instinct, tolerance of negative affect, and strengthening effects of stress), acceptance (i.e., positive acceptance of change and secure relationships), control (i.e., sense of control), and spirituality (i.e., spiritual influences) [57, 74]. This study calculated the composite scores for each factor by aggregating corresponding items, and Cronbach’s α of tenacity, tolerance, acceptance, control and spirituality were 0.865, 0.860, 0.798, 0.736, and 0.419, respectively.

### Analytic approach

All statistical analyses were performed using IBM SPSS 26.0 and Mplus 8.3. The statistical significance level was set at a two-tailed 0.05. First, descriptive statistics were calculated for demographic characteristics. Second, the direct effects of COVID-19 information exposure on psychological disorders were examined and demographic variables (i.e., age, sex, ethnic group, and education level) were controlled as covariates. Third, a latent mediation model was applied by controlling the demographic variables to investigate mediating effects by using latent COVID-19 information exposure as the independent variable, latent risk perception as the mediator, and latent emotional distress as the dependent variable. Finally, with the demographics controlled as covariates, a latent moderated mediation model was constructed to investigate the latent interactions of resilience and risk perception on emotional distress, and the changes of risk perception’s mediating effects.

Two steps were needed to test the moderating effects of resilience. First, a benchmark null model (i.e., Model 0) was built to assess the moderating effects of resilience only on emotional distress. Model fitness was evaluated using the chi-squared-degree of freedom ratio (*χ*^*2*^*/*df), comparative fit index (CFI), Tucker–Lewis index (TLI), root mean square error of approximation (RMSEA), and standardized root mean residual (SRMR) [77]. The acceptable criteria for the model were set as CFI > 0.90, TLI > 0.90, RMSEA < 0.08, and SRMR < 0.08 [78]. Next, Model 1 was constructed by adding latent interactions (i.e., risk perception and resilience) based on Model 0. A log-likelihood ratio test was conducted to examine whether Model 1 was better than Model 0. Model 1 would be deemed to better fit the data than Model 0 if the log-likelihood ratio test produced a significant value and the latent interactions could significantly predict emotional distress.

## Results

### Demographic characteristics

The majority of the 1313 participants (*M*_age_ = 19.76 ± 2.25 years) were female (73.1%), Han ethnic (85.1%), and undergraduate students (94.6%). The reported frequency of exposure to COVID-19 information was 5.73 (SD = 4.18) times per day, and the exposure duration was 1.46 (SD = 1.18) hours per day (see Table 1).

**Table 1:** Sociodemographic characteristics of the sample (*N* = 1313).

Variable	Mean (SD) or *n* (%)
Age	19.76 (2.25)
Sex	
Male	353 (26.9%)
Female	960 (73.1%)
Ethnic group	
Han	1118 (85.1%)
Others	195 (14.9%)
Education	
Undergraduate	1242 (94.6%)
Graduate	71 (5.4%)
COVID-19 information exposure	
Frequency (times per day)	5.73 (4.18)
≤1	123 (7.6%)
2	142 (10.8%)
3	179 (13.6%)
4	180 (13.7%)
5	185 (14.1%)
6	96 (7.3%)
7	30 (2.3%)
8	125 (9.5%)
9	44 (3.4%)
10	82 (6.2%)
≥11	127 (9.7%)
Duration (hours per day)	1.46 (1.18)
≤1	912 (69.5%)
2	274 (20.9%)
3	75 (5.7%)
4	24 (1.8%)
5	12 (0.9%)
≥6	16 (1.2%)

### Common method bias and correlations between main variables

The use of same-source data for independent and dependent variables might introduce the possibility of common method bias in the present study. Therefore, we conducted Harman’s single factor test [79] to examine the common method bias. The results showed that the single un-rotated factor only explained 33.9% of the variance, indicating that common method bias was not a major problem in this study.

The results of the correlation analysis revealed that the frequency (*r*_anxiety_ = 0.19, *p* = 0.000; *r*_depression_ = 0.14, *p* = 0.000) and the duration (*r*_anxiety_ = 0.17, *p* = 0.000; *r*_depression_ = 0.12, *p* = 0.000) of COVID-19 information exposure were positively associated with anxiety and depression (see Table 2).

**Table 2:** Pearson correlation between main variables (*N* = 1313).

Variable	*M*	*SD*	1	2	3	4	5	6	7	8	9
1. Age	19.76	2.25	1								
2. Sex (male)	–		−0.01	1							
3. Ethnicity (Han)	–		0.05	0.00	1						
4. Education level	–		0.83***	−0.07*	0.12***	1					
5. Exposure frequency	5.73	4.18	−0.06*	−0.02	−0.01	−0.04	1				
6. Exposure duration	1.46	1.18	0.00	−0.01	−0.00	−0.00	0.33***	1			
7. Perceived risk	3.24	1.93	−0.03	−0.06*	−0.03	−0.03	0.09**	0.03	1		
8. Resilience	93.38	13.60	−0.06*	0.02	0.06*	−0.08**	−0.03	−0.05	−0.10***	1	
9. Anxiety	9.78	3.48	0.14***	−0.07**	0.00	0.19***	0.19***	0.17***	0.22***	−0.32***	1
10. Depression	12.57	4.24	0.10***	0.02	−0.03	0.14***	0.14***	0.12***	0.23***	−0.39***	0.71***

Sex, ethnicity and education level were coded as dummy variables (i.e., male = 1, female = 0; Han ethnic group = 1, others = 0; undergraduate = 1, graduate = 0).

*p < 0 .05, **p < 0 .01, ***p < 0 .001.

### Effects of COVID-19 information exposure

The direct effects of COVID-19 information exposure on emotional distress were examined using a model with information exposure (i.e., frequency and duration) as the latent independent variable and emotional distress (i.e., anxiety and depressive symptoms) as the latent dependent variable. The correlation results showed that some demographic variables (i.e., age, sex, and education level) were correlated with the independent, dependent or mediating variables. Therefore, these demographics were controlled as covariates in the subsequent models. This model fit well with the data (*χ*^*2*^*/*df = 1.858, CFI = 0.993, TLI = 0.987, RMSEA = 0.026, 90% CI = [0.003, 0.043], SRMR = 0.018). The results showed that increased exposure to COVID-19 information predicted greater emotional distress (*β* = 0.30, 95% CI = [0.20, 0.39], *p* = .000).

**Fig. 1:**
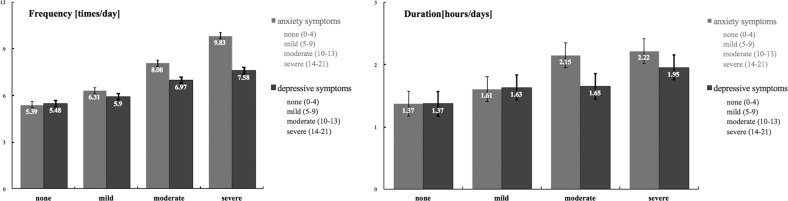
The severity of anxiety and depressive symptoms with different degrees of COVID-19 information exposure. The numbers on the histogram represent the means of the frequency or duration of COVID-19 information exposure; the error bars represent the standard errors.

Further, the relationships between the frequency and duration of information exposure and the severity of anxiety and depressive symptoms were examined through descriptive means. As Fig. 1 shows, the critical threshold of seven times or 2 h per day was obtained to mark the difference between mild and moderate anxiety symptoms; the critical value of six times or 1.64 h (38 min) per day was attained to differentiate between mild and moderate depressive symptoms.

### Mediating effects of perceived risk

**Fig. 2:**
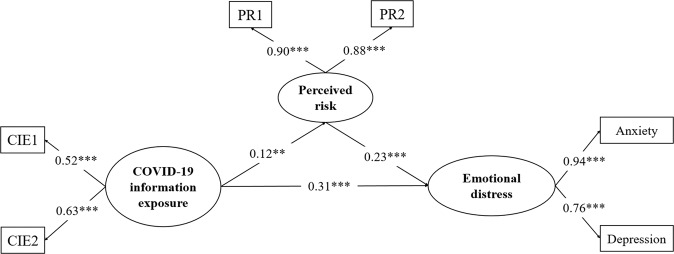
The latent mediation model. CIE1 and CIE2 denote items measuring COVID-19 information exposure; PR1 and PR2 denote items measuring perceived risk. **p < 0.01, ***p < 0.001.

The mediating effects of perceived risk were examined through a latent mediation model (see Fig. 2), which evinced a good fit with the data (*χ*^*2*^*/*df = 2.180, CFI = 0.990, TLI = 0.985, RMSEA = 0.030, 90% CI = [0.018, 0.042], SRMR = 0.022). The results evidenced that COVID-19 information exposure predicted higher perceived risk (*β* = 0.12, 95% CI = [0.05, 0.19], *p* = .005), which projected severer emotional distress (*β* = 0.23, 95% CI = [0.18, 0.28], *p* = 0.000). In brief, the results of the indirect effects demonstrated that perceived risk significantly mediated the effects of COVID-19 information exposure on emotional distress (*β* = 0.03, 95% CI = [0.01, 0.04], *p* = 0.005).

### Moderating effects of resilience

**Fig. 3:**
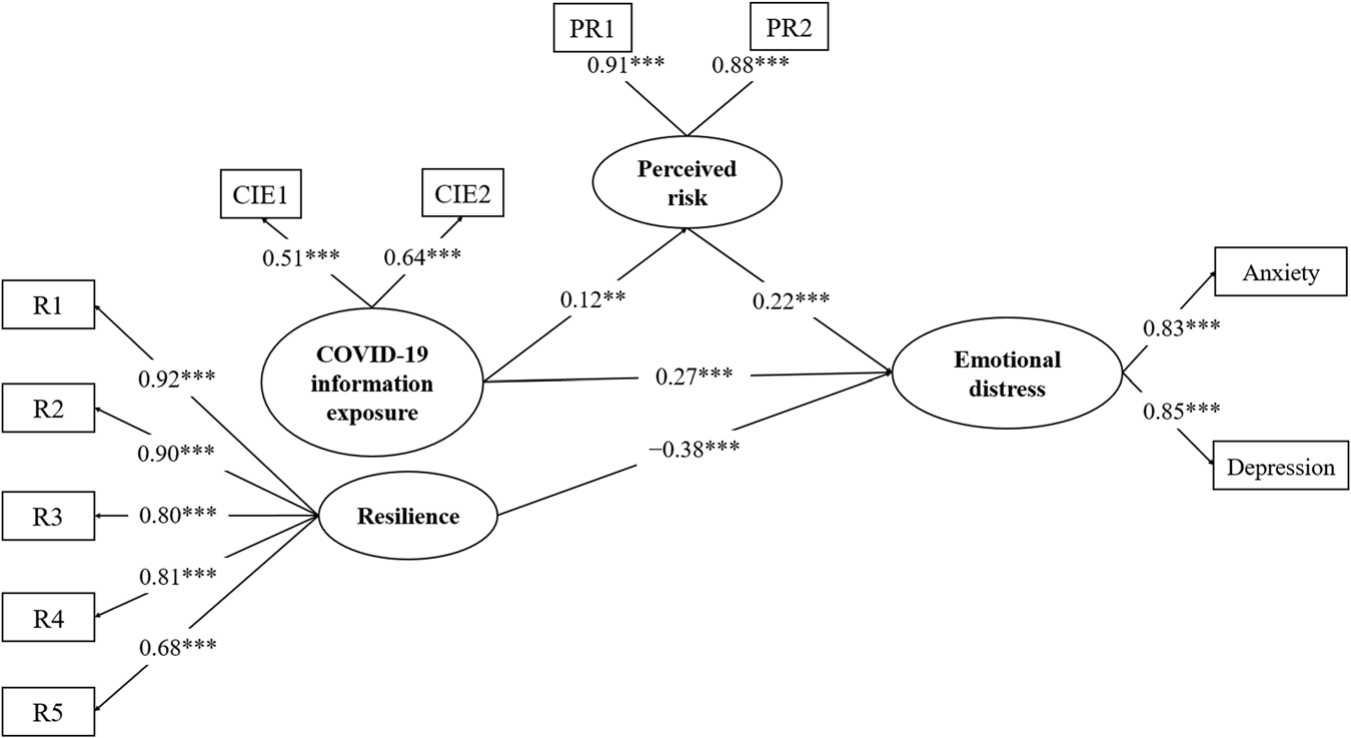
The null model without estimation of latent interactions. CIE1 and CIE2 denote items measuring COVID-19 information exposure; PR1 and PR2 denote items measuring perceived risk; RE1–RE5 indicate the five factors of resilience. **p < 0.01, ***p < 0.001.

**Fig. 4:**
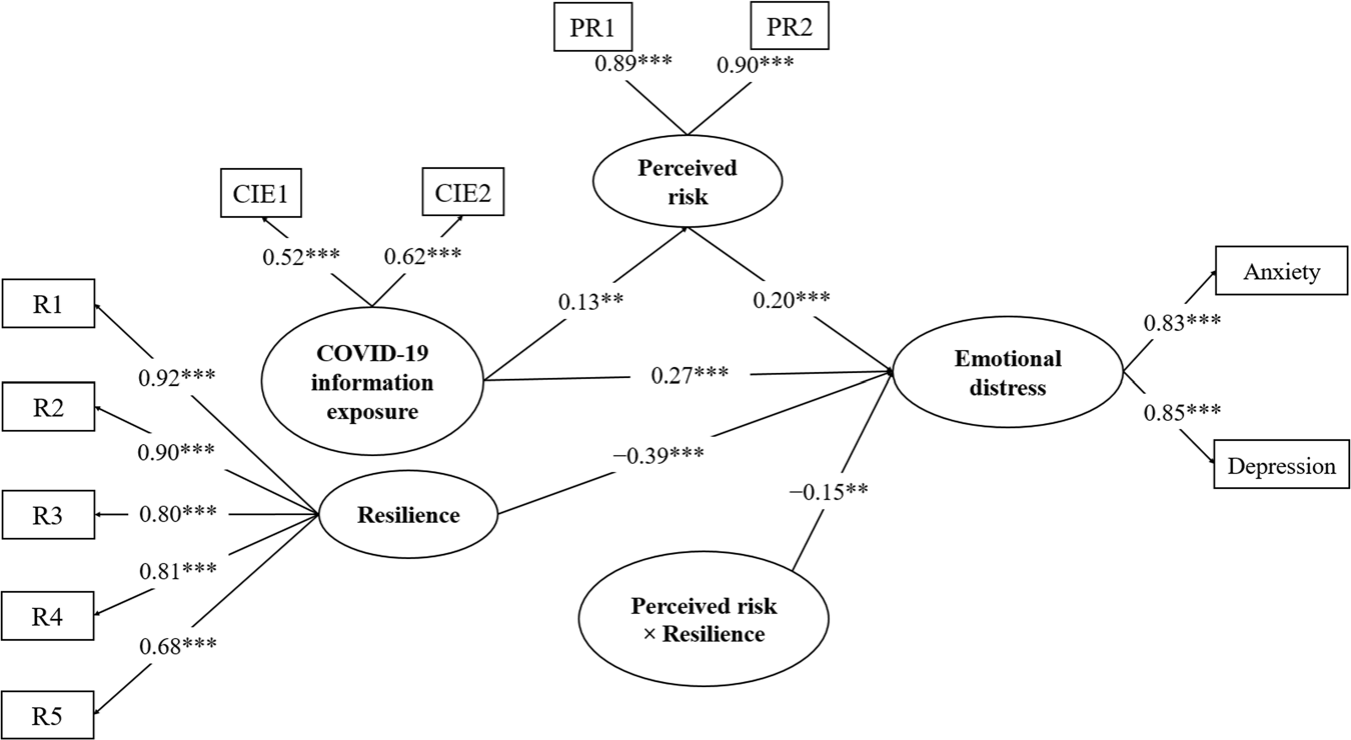
The latent moderated mediation model (the Model 1). CIE1 and CIE2 denote items measuring COVID-19 information exposure; PR1 and PR2 denote items measuring perceived risk; RE1–RE5 indicate the five factors of resilience. **p < 0.01, ***p < 0.001.

The moderating effects of resilience were examined using a latent moderated mediation model. First, Model 0 (see Fig. 3) without latent interactions presented a good fit (*χ*^*2*^*/*df = 4.976, CFI = 0.964, TLI = 0.954, RMSEA = 0.055, 90% CI = [0.049, 0.061], SRMR = 0.040). Second, Model 1 (see Fig. 4) including the latent interactions of risk perception and resilience showed a significant log-likelihood ratio value (*D* = 19.266, df = 1, *p* = 0.000). Thus, Model 1 fit the data better than Model 0.

**Fig. 5:**
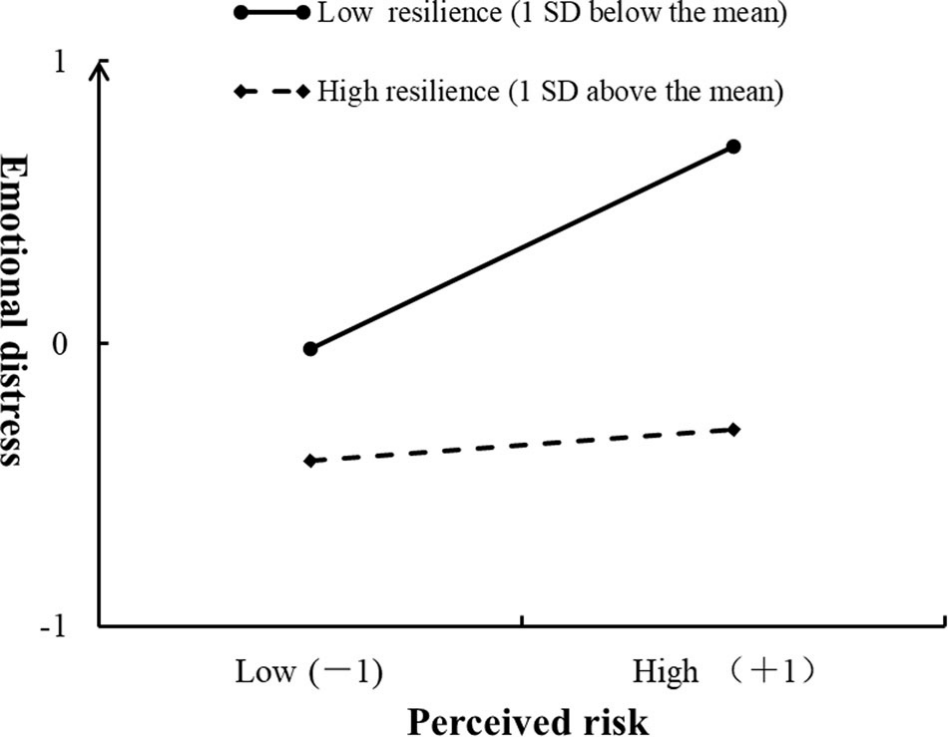
The simple slope analysis for the moderating effects of resilience. The ±1 on the horizontal axis represents the perceived risk at 1 SD above and below the mean.

Figure 4 displays that resilience moderated the mediating effects of risk perception, as indicated by the significant interaction between perceived risk and resilience on emotional distress (*β* = −0.15, 95% CI = [−0.22, −0.07], *p* = 0.002). Moreover, the differences between the mediating effects of risk perception were analyzed at different levels of resilience. The results revealed that the indirect effects of risk perception at a high level (1 SD above the mean) of resilience (*β* = 0.01, 95% CI = [−0.01, 0.02]) were weaker than those at a low level (1 SD below the mean) of resilience (*β* = 0.08, 95% CI = [0.01, 0.13]). The results indicate that the mediating effects of risk perception between COVID-19 information exposure and emotional distress diminish with an increase in resilience. Specifically, Fig. 5 illustrates that greater perceived risk predicted severer emotional distress (*β* = 0.34, 95% CI = [0.25, 0.44], *p* = 0.000) at low levels of resilience. However, the prediction of risk perception on emotional distress decreased at high resilience levels (*β* = 0.06, 95% CI = [−0.03, 0.14], *p* = 0.276).

The present study also tested the moderating effects of each factor of psychological resilience to investigate the specific roles of each factor on the prevention of emotional distress. The results showed that all factors except for tolerance significantly moderated the mediating effects of risk perception on emotional distress (*β* = −0.07, 95% CI = [−0.19, 0.05], *p* = 0.365) (see Figs. S2–S10 in supplementary materials). The moderating effects of tenacity (*β* = −0.15, 95% CI = [−0.23, −0.07], *p* = 0.002), acceptance (*β* = −0.14, 95% CI = [−0.25, −0.03], *p* = 0.033), control (*β* = −0.11, 95% CI = [−0.19, −0.02], *p* = 0.048), and spirituality (*β* = −0.17, 95% CI = [−0.27, −0.08], *p* = 0.004) were particularly significant.

## Discussion

This study investigated the effects of COVID-19 information exposure on mental health during the pandemic, particularly in China. It clarified the relationships between information exposure and mental health with respect to risk perception and psychological resilience. It confirmed previous studies’ findings that overexposure to COVID-19 information amplifies emotional distress and supported the conclusion that the infodemic could negatively impact mental health. Overexposure to COVID-19 information may spark an overload of information and a surge of fake news. Thresholds of COVID-19 information exposure were identified as 7 times or 2 h per day for moderate anxiety symptoms and six times or 38 min per day for depressive symptoms. Notably, critical threshold values in this study were lower than those found in other countries [8, 20, 21], indicating that the Chinese public may suffer severer emotional distress given the same amount of information exposure. The possible explanation is that Chinese people are more collectivistic [80] and more relying on situational signs [81]; thus, they are possibly more sensitive to the pandemic context and related information, exhibiting more anxiety or depression especially when they were social-isolated.

As hypothesized, risk perception partially mediated the association between COVID-19 information exposure and emotional distress. Overloading of COVID-19 information or misinformation may cause inaccurate risk perception, thus inducing anxiety or depressive symptoms [21, 82]. The mediating effects of risk perception were moderated by psychological resilience, which buffered the negative effects of COVID-19 information exposure on mental health, indicating that low resilience entails vulnerability to emotional distress from the high perceived risk of COVID-19. Furthermore, the resilience factors (i.e., tenacity, acceptance, control, and spirituality) protect individual mental health against the threat of high-risk perception: not all five resilience factors equally safeguard against risk perception [83]. This result suggests that handling unpleasant feelings or tolerating adverse circumstances is not enough to fight an infodemic; rather, individuals must respond actively and adapt optimistically.

This study has theoretical and practical implications. It is among the first to illuminate the mediating role that risk perception plays in the association between information exposure and emotional distress during the COVID-19 pandemic [40]. It also investigated the role of resilience, thereby providing an expanded framework for the association between COVID-19 information exposure and mental health. Moreover, this study is the first to evaluate the five factors of resilience separately and demonstrate their disparate roles in preventing emotional distress, thereby resolving long-standing concerns regarding the construct of resilience [53] and enriching the theory of psychological resilience.

Furthermore, this study provides critical practical insights for the public, psychiatrists and government in the pandemic. First, for the public, this study shows that information overload should be reduced by limiting the frequency and duration of accessing COVID-19-related information [84]. People should also rely more on updated authentic information from authoritative media sources, such as the WHO and other official entities, than social media such as WeChat or blogs [23, 85]. Second, for the psychiatrists, psychologists, and public health workers, our data established a distinct threshold for COVID-19 information exposure associated with risk of anxiety or depressive symptoms. This cutoff can be a reference for clinical psychiatric assessments [8]. Furthermore, psychiatrists and psychologists should deliver mental health knowledge, resources, and services through mainstream media. For instance, online psychological counseling services were afforded in some Chinese cities during the COVID-19 outbreak [86, 87] and were found to help a group of insomnia sufferers [18]. Moreover, this study showed that interventions against the effects of COVID-19 information overexposure must target psychological resilience. Resilience is not a trait but ideas and behaviors that can be learned and developed [88]. Psychologists and psychiatrists could educate the public about ways of building and developing resilience [54]. Specifically, developing certain sub-dimensional abilities within resilience, such as tenacity, acceptance, and spirituality, is necessary. Lastly, for policymakers, this study indicates that official agencies should regulate COVID-19-related information dissemination. Previous studies have indicated that misinformation restricts governments from effectively responding to crises [16, 89]; thus, governments must release unified and accurate information in a timely manner through official media channels [23]. They should also reduce the overexposure of susceptible individuals to pandemic-related information, especially duplicate negative information. Governments could also provide positive information on preventive or protective measures to offset negative information on the pandemic, which could increase individual resilience against heightened risk perception.

The study acknowledges some limitations. First, only students skilled in mobile phone use were recruited, and the sample may not be representative of users of legacy media. Second, this study only examined the amount (i.e., frequency and duration) of information exposure. Several studies have found that the content of the information also affect mental health [90]. Thus, future surveys may also include information content. Third, this study found that risk perception partially mediated the relationship between COVID-19 information and emotional distress, whereas previous studies found that other factors, such as perceived self-efficacy [47] and information processing modes [33, 91], also influence the association. Thus, it is necessary to identify the mechanism of information exposure’s effects on mental health more comprehensively. Fourth, this study used a cross-sectional design and only collected self-reported indicators. Future research could adopt objective biological indicators, such as peripheral blood heredity, immune and metabolic function markers, cerebrospinal fluid indicators, cortisol, or brain imaging. Finally, although this study was conducted when the pandemic broke out, the investigation time provided a comprehensive reference for different pandemic stages in China, including the outbreak, peak, and after peak stage in 2020. More research during the after peak period is required to examine the robustness of the results.

In conclusion, this study found that overexposure to COVID-19 information in China has increased individual perceived risk and thus amplified emotional distress. Further, it confirmed the buffering role of resilience for mitigating perceived risk and is the first to investigate the moderating effects of five resilience sub-factors. The development of psychological resilience, especially tenacity, acceptance, control, and spirituality, could be an antidote to anxiety and depressive symptoms in an infodemic.

## Supplementary information

Supplemental Materials

## Data Availability

The datasets used in the present study are available from the corresponding author on reasonable request.

## Materials availability

The materials used in the present study are available from the corresponding author on reasonable request.

## Code availability

The codes used in the present study are available from the corresponding author on reasonable request.
