# Supplementary material for: Overexposure to COVID-19 information amplifies emotional distress: a latent moderated mediation model
Source: Transl Psychiatry. 2022 Jul 18;12:287. doi: 10.1038/s41398-022-02048-z (PMC9293900; doi:10.1038/s41398-022-02048-z)
Supplement: Supplementary file 1 — Supplemental Materials [file 41398_2022_2048_MOESM1_ESM.docx]

**Overexposure to COVID-19 information amplifies emotional distress: A latent moderated mediation model**

Supplemental Materials

**Data collection period**

We collected data from February 2 to March 3, 2020, during which the number of infected patients kept increasing and the government issued nationwide self-isolation and social-isolation policies. Figure 1S shows the period of this survey and the situation in Chinese mainland regarding the COVID-19 pandemic.

**
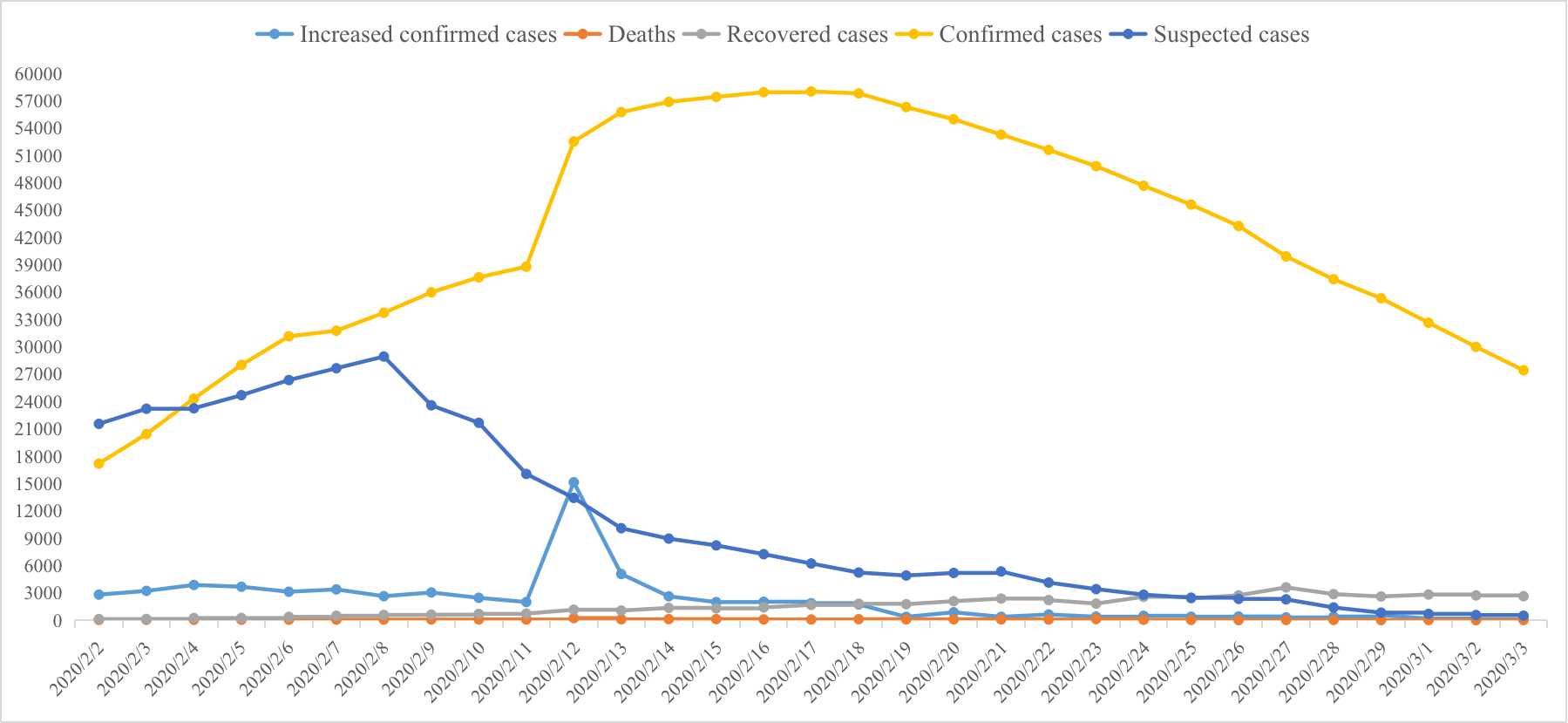
**

**Figure S1.** Timeline of the COVID-19 pandemic situation in Chinese mainland during the data collection period

*Note.* Data source: National Health Commission of the People’s Republic of China, <http://www.nhc.gov.cn>.

**Additional Results**

In order to further investigate the effects of resilience on emotional distress, we examined the moderation of five resilience factors one by one in this study. Similar to the moderating effect of resilience, we conducted five latent moderated mediation models to examine the moderating effects of each resilience factor.

## Moderating effects of tenacity

The Factor 1 (i.e., tenacity) of resilience represents the notion of personal competence, high standards, and tenacity (Connor & Davidson, 2003). It consists of eight items, for example, “You work to attain your goals”, “When things look hopeless, I don’t give up”. The internal consistency of this subscale was good (Cronbach’s α = 0.865) in this study.

A latent moderated mediation model was used to examine the moderating effect of tenacity. First, the Model 0 without latent interaction showed a good fit (*χ^2^/df* = 7.611, CFI = 0.909, TLI = 0.889, RMSEA = 0.071, 90% *CI* = [0.066, 0.076], SRMR = 0.047). Second, the Model 1 (see *Figure S2*) with latent interaction (i.e., perceived risk×tenacity) showed a significant log likelihood ratio value (D = 20.012, *df* = 1, *p* = .000), indicating that Model 1 fit the data better than Model 0.

*
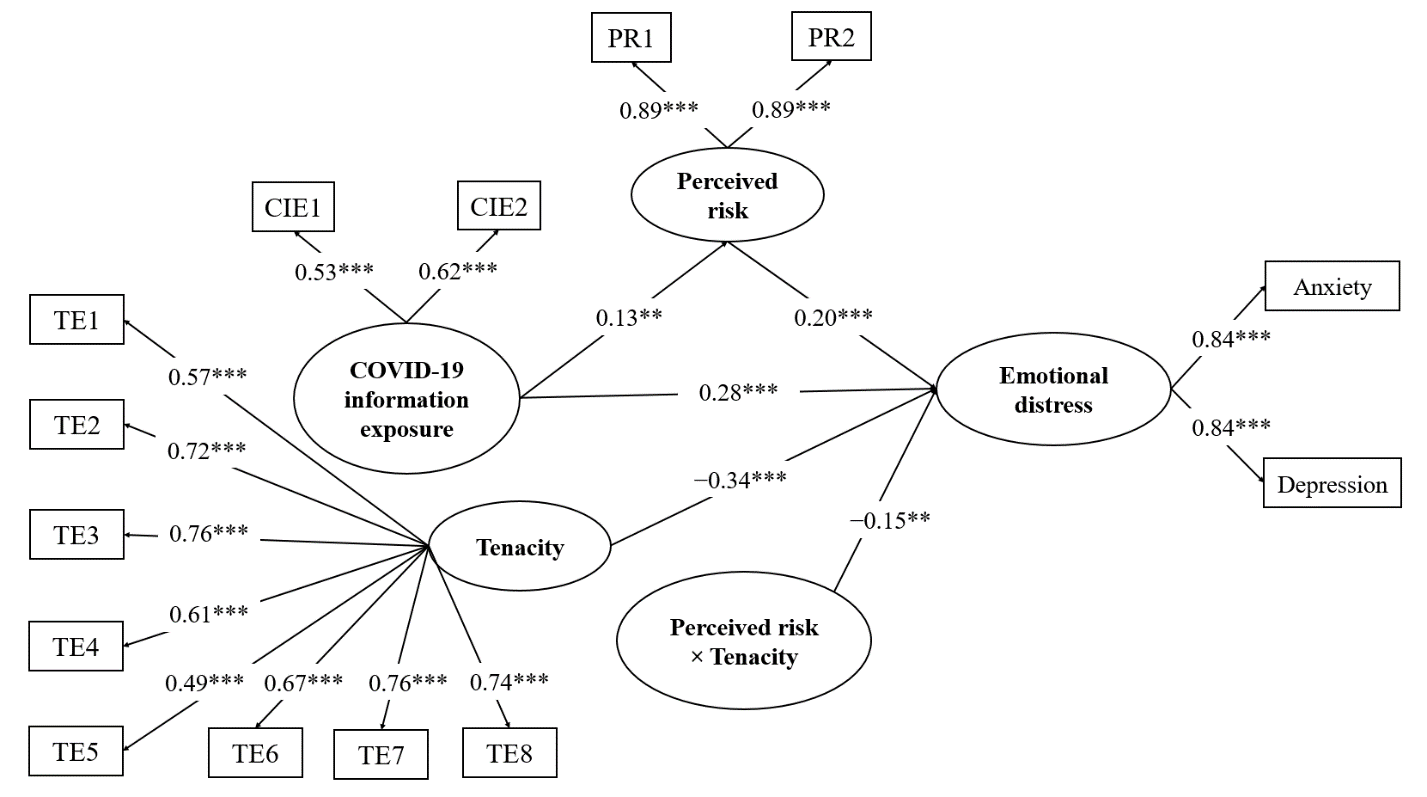
*

**Figure S2.** The latent moderated mediation model with tenacity as moderator

*Note*. CIE1 and CIE2 denote items measuring COVID-19 information exposure; PR1 and PR2 denote items measuring perceived risk; TE1-TE8 indicate the tenacity subscale. ***p* < .01, ****p* < .001.

Model 1 showed that tenacity moderated the mediating effects of risk perception by a significant interaction between perceived risk and tenacity on emotional distress (*β* = −0.15, 95% *CI* = [−0.23, −0.07], *p* = .002), as indicated in Figure S2. Moreover, we analyzed the difference of mediating effects of risk perception at different levels of tenacity. The results showed that the indirect effect of risk perception at high level (1 *SD* above the mean) of tenacity (*β* = −0.01, 95% *CI* = [−0.05, 0.02]) is weaker than that at low level (1 *SD* below the mean) of tenacity (*β* = 0.10, 95% *CI* = [0.01, 0.18]), indicating that with the increase of tenacity, the mediating effects of risk perception between COVID-19 information exposure and emotional distress reduced. Specifically, as shown in Figure S3, at low levels of tenacity, greater perceived risk predicted severer emotional distress (*β =* 0.45, 95% *CI* = [0.31, 0.58], *p* = .000). However, at high levels of tenacity, the prediction of risk perception on emotional distress is not significant (*β* = −0.05, 95% *CI* = [−0.17, 0.08], *p* = .516).

**
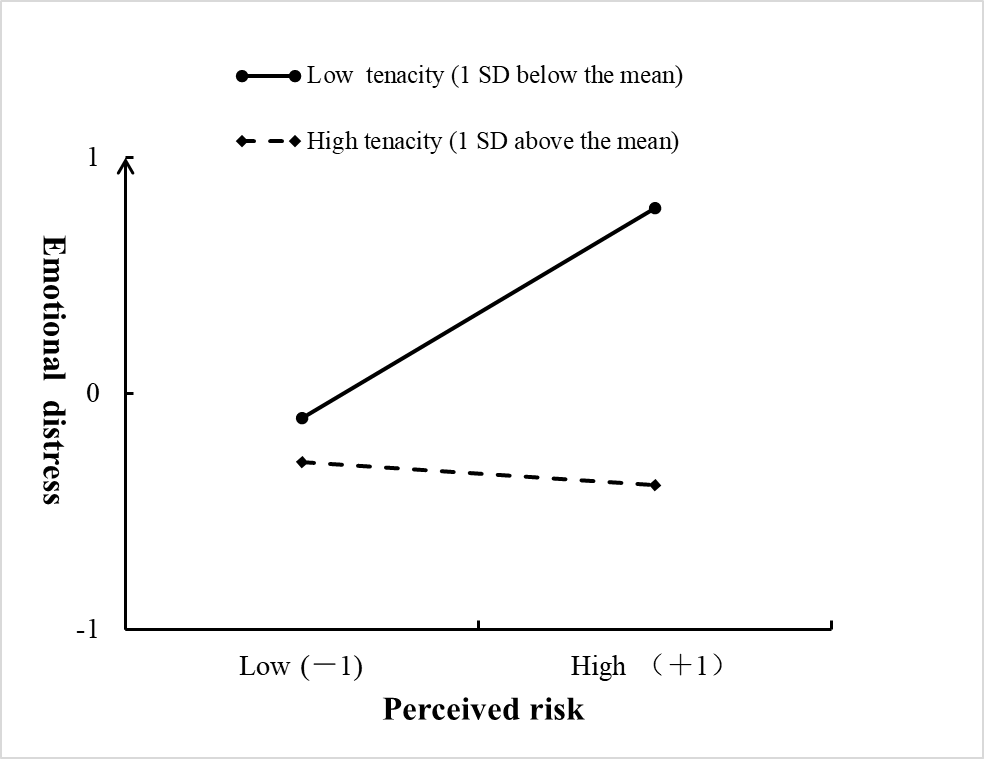
**

**Figure S3.** The simple slope analysis for the moderating effects of tenacity

*Note*. The dash line represents that the prediction of perceived risk on emotional distress is not significant.

## Moderating effects of tolerance

The Factor 2 (i.e., tolerance) of resilience relates to the trust in one’s instincts, tolerance of negative affect and strengthening effects of stress (Connor & Davidson, 2003). It consists of seven items, for instance, “Have to act on a hunch”, “Can handle unpleasant feelings”, and “Make unpopular of difficult decisions”. The internal consistency of this subscale was good (Cronbach’s α = 0.860) in this study.

As for the moderating effect of tolerance, first, the Model 0 without latent interaction showed a good fit (*χ^2^/df* = 3.728, CFI = 0.963, TLI = 0.954, RMSEA = 0.046, 90% *CI* = [0.041, 0.051], SRMR = 0.037). Second, the Model 1 (see *Figure S4*) with latent interaction (i.e., perceived risk×tolerance) showed a non-significant log likelihood ratio value (D = 5.204, *df* = 1, *p* = .000). This indicated that Model 1 did not fit the data better than Model 0. Besides, in Model 1, the interaction effects of perceived risk and tolerance on emotional distress was not significant (*β* = −0.07, 95% *CI* = [−0.19, 0.05], *p* = .365). Thus, the tolerance factor did not moderated the mediating effects of risk perception between COVID-19 information exposure and emotional distress. Despite of the absence of moderating effects, tolerance showed a main effect on emotional distress, that is, higher tolerance predicted lower emotional distress (*β* = −0.42, 95% *CI* = [−0.48, −0.36], *p* = .000).

*
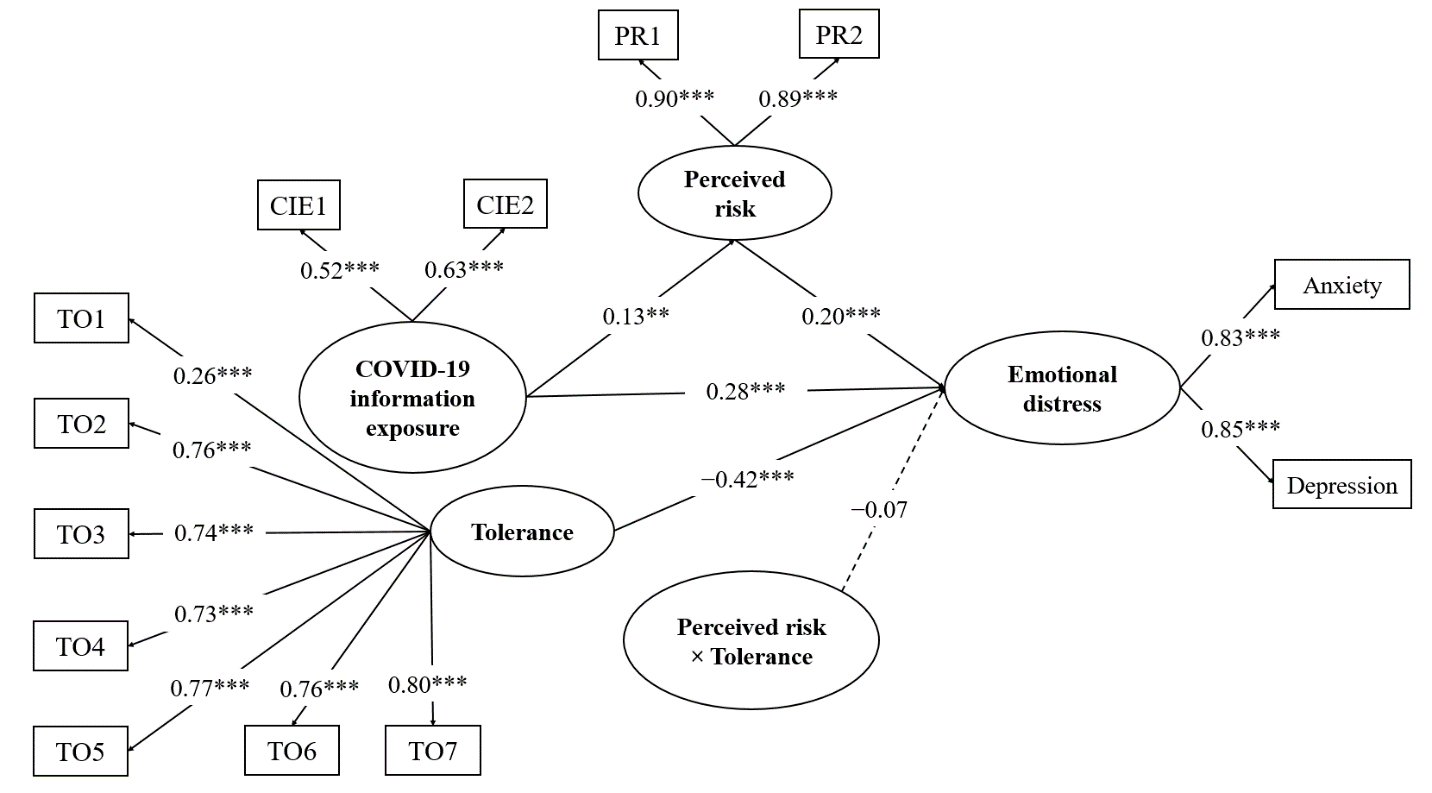
*

**Figure S4.** The latent moderated mediation model with tolerance as moderator

*Note*. CIE1 and CIE2 denote items measuring COVID-19 information exposure; PR1 and PR2 denote items measuring perceived risk; TO1-TO7 indicate the tolerance subscale. The dash line represents that the predictive path is not significant. ***p* < .01, ****p* < .001.

## Moderating effects of acceptance

The Factor 3 (i.e., acceptance) of resilience reflects the positive acceptance of change and secure relationships (Connor & Davidson, 2003), corresponding to the notion of self-esteem (Haddadi & Besharat, 2010). Five items consisted this subscale. Example items include “Able to adapt to change”, “Can deal with whatever comes”. We deleted the item 8 (i.e., “Tend to bounce back after illness or hardship”) due to its extreme low factor loading of 0.007, according to the results of exploratory factor analysis. The internal consistency of the remaining four items was good (Cronbach’s α = 0.802) in this study.

The moderating effect of acceptance was examined by a latent moderated mediation model. First, the Model 0 without latent interaction showed a good fit (*χ^2^/df* = 3.469, CFI = 0.962, TLI = 0.952, RMSEA = 0.043, 90% *CI* = [0.037, 0.049], SRMR = 0.037). Second, the Model 1 (see *Figure S5*) with latent interaction (i.e., perceived risk×acceptance) showed a significant log likelihood ratio value (D = 13.61, *df* = 1, *p* = .000), indicating that Model 1 had better fitness with the data than Model 0.

*
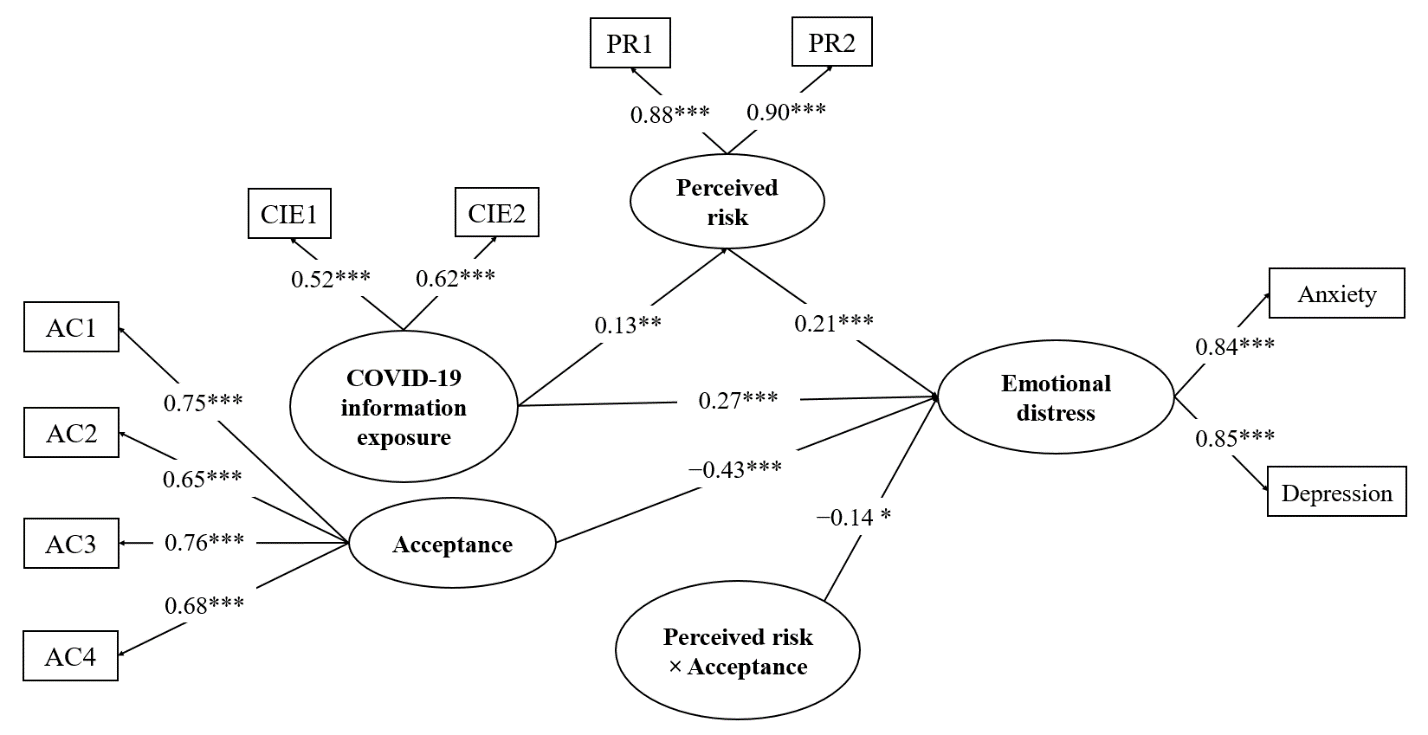
*

**Figure S5.** The latent moderated mediation model with acceptance as moderator

*Note*. CIE1 and CIE2 denote items measuring COVID-19 information exposure; PR1 and PR2 denote items measuring perceived risk; AC1-AC4 indicate the acceptance subscale. **p* < .05, ***p* < .01, ****p* < .001.

As illustrated in Figure S5, the results showed that acceptance moderated the mediating effects of risk perception by a significant interaction between perceived risk and acceptance on emotional distress (*β* = −0.14, 95% *CI* = [−0.25,−0.03], *p* = . 033). Furthermore, the difference of mediating effects of risk perception at different levels of acceptance was calculated, showing that the indirect effect of risk perception at high level (1 *SD* above the mean) of acceptance (*β* = 0.01, 95% *CI* = [−0.03, 0.04]) is weaker than that at low level (1 *SD* below the mean) of acceptance (*β* = 0.08, 95% *CI* = [0.01, 0.15]). This suggested that the mediating effects of risk perception between COVID-19 information exposure and emotional distress reduced with the increase of acceptance. In detail, as shown in Figure S6, at low levels of acceptance, greater perceived risk predicted severer emotional distress (*β =* 0.39, 95% *CI* = [0.25, 0.52], *p* = .000). However, at high levels of acceptance, the prediction of risk perception on emotional distress is not significant (*β =* 0.04, 95% *CI* = [−0.12, 0.20], *p* = .680).

*
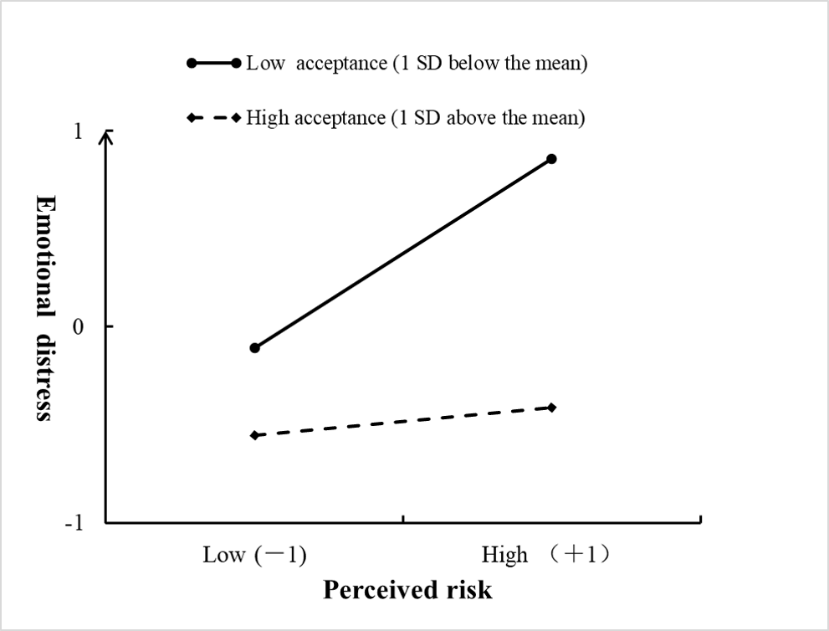
*

**Figure S6.** The simple slope analysis for the moderating effects of acceptance

*Note*. The dash line represents that the prediction of perceived risk on emotional distress is not significant.

## Moderating effects of control

The Factor 4 (i.e., control) of resilience refers to the sense of control (Connor & Davidson, 2003). Three items constitutes this subscale, that is, “In control of your life”, “Know where to turn for help” and “Strong sense of purpose”. The internal consistency of this subscale was acceptable (Cronbach’s α = 0.736) in this study.

A latent moderated mediation model was built to investigate the moderating effect of control. First, the Model 0 without latent interaction showed a good fit (*χ^2^/df* = 3.512, CFI = 0.969, TLI = 0.958, RMSEA = 0.044, 90% *CI* = [0.037, 0.051], SRMR = 0.037). Second, the Model 1 (see *Figure S7*) with latent interaction (i.e., perceived risk×control) showed a significant log likelihood ratio value (D = 7.528, *df* = 1, *p* = .000), indicating that Model 1 fit the data better than Model 0.

*
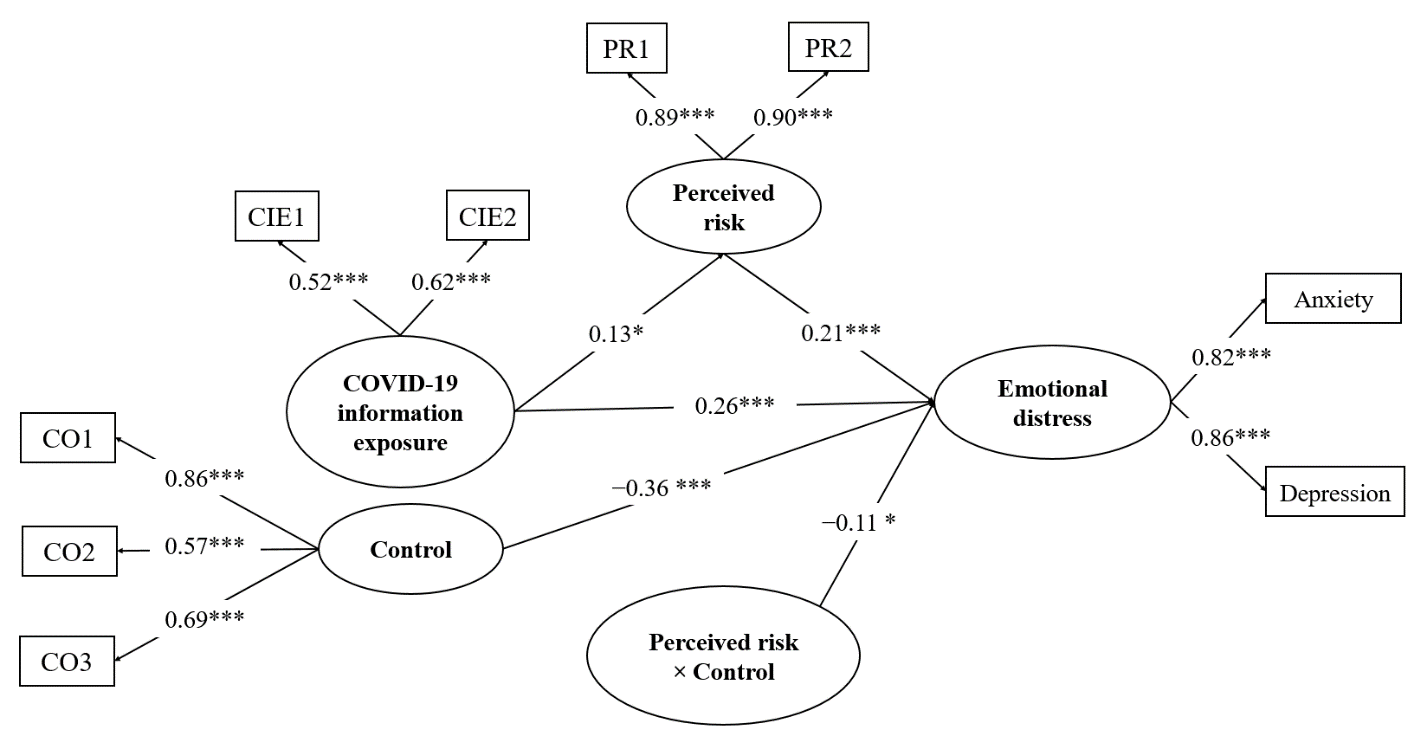
*

**Figure S7.** The latent moderated mediation model with control as moderator

*Note*. CIE1 and CIE2 denote items measuring COVID-19 information exposure; PR1 and PR2 denote items measuring perceived risk; CO1-CO3 indicate the control subscale. **p* < .05, ****p* < .001.

As shown in Figure S7, control moderated the mediating effects of risk perception by a significant interaction between perceived risk and control on emotional distress (*β* = −0.11, 95% *CI* = [−0.19, −0.02], *p* = .048). Moreover, the mediating effects analysis of risk perception showed that the indirect effect of risk perception at high level (1 *SD* above the mean) of control (*β* = 0.02, 95% *CI* = [−0.10, 0.04]) is weaker than that at low level (1 *SD* below the mean) of control (*β* = 0.07, 95% *CI* = [0.01, 0.13]), indicating that with the increase of control, the mediating effects of risk perception between COVID-19 information exposure and emotional distress weakened. To be specific, as shown in Figure S8, at low levels of control, greater perceived risk predicted severer emotional distress (*β =* 0.32, 95% *CI* = [0.22, 0.43], *p* = .000). However, at high levels of control, the prediction of risk perception on emotional distress is not significant (*β =* 0.07, 95% *CI* = [−0.02, 0.16], *p* = .193).

*
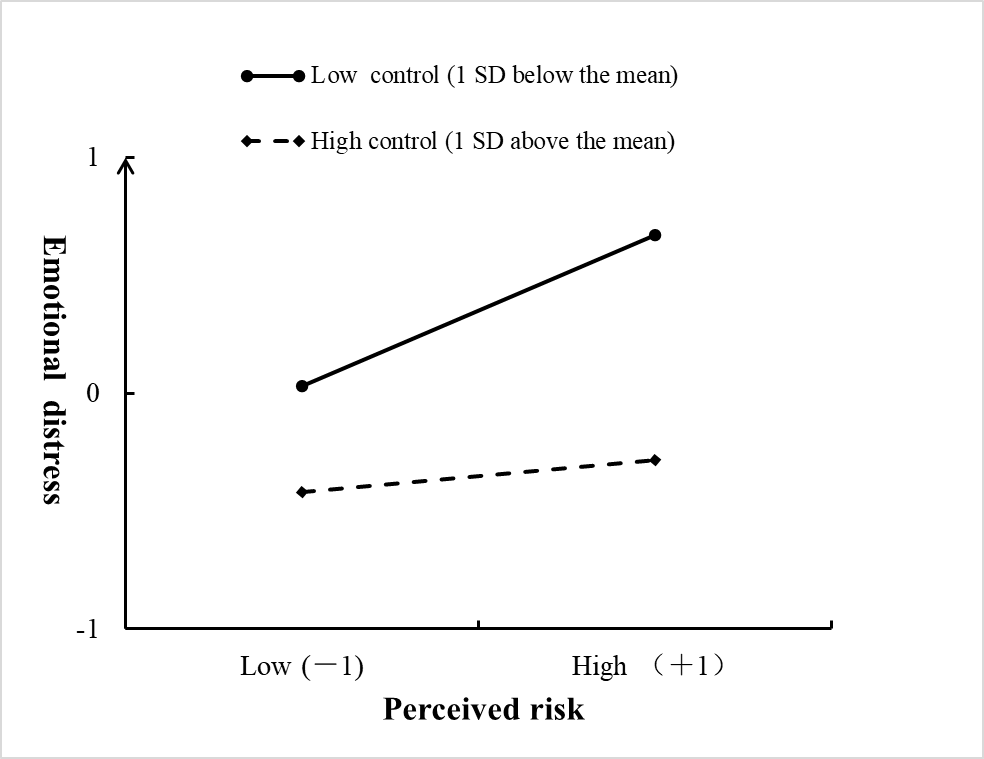
*

**Figure S8.** The simple slope analysis for the moderating effects of control

*Note*. The dash line represents that the prediction of perceived risk on emotional distress is not significant.

## Moderating effects of spirituality

The Factor 5 (i.e., spirituality) of resilience relates to the spiritual influences (Connor & Davidson, 2003). It consists of two items: “Sometimes fate or God can help” and “Things happen for a reason”. A composite score of spirituality (Cronbach’s α = 0.419) was calculated in this study.

A latent moderated mediation model was built to test the moderating role of spirituality factor, the same as above four factors. First, the Model 0 without latent interaction showed a good fit (*χ^2^/df* = 2.216, CFI = 0.984, TLI = 0.977, RMSEA = 0.030, 90% *CI* = [0.021, 0.040], SRMR = 0.027). Second, the Model 1 (see *Figure S9*) with latent interaction (i.e., perceived risk×spirituality) showed a significant log likelihood ratio value (D = 15.366, *df* = 1, *p* = .000), indicating that Model 1 fit the data better than Model 0.

*
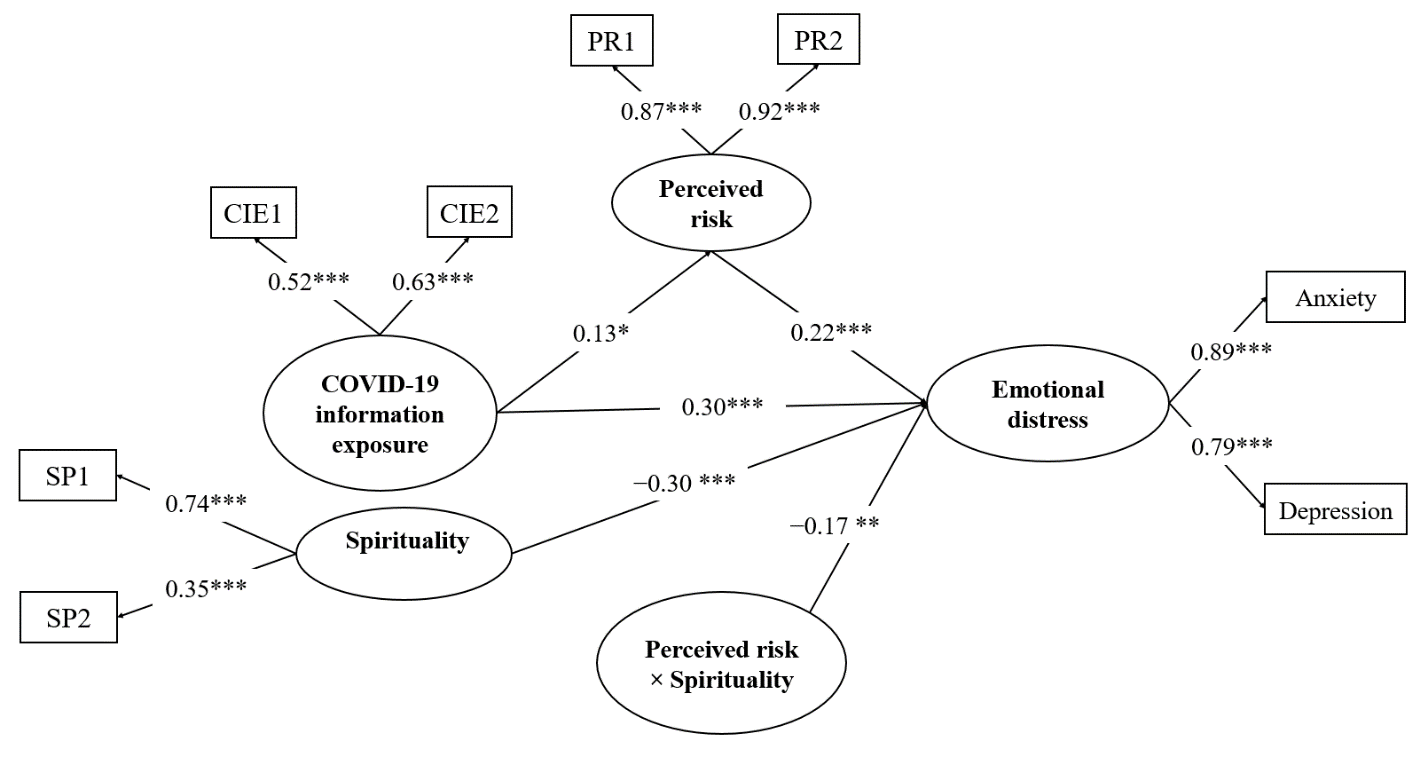
*

**Figure S9.** The latent moderated mediation model with spirituality as moderator

*Note*. CIE1 and CIE2 denote items measuring COVID-19 information exposure; PR1 and PR2 denote items measuring perceived risk; SP1 and SP2 indicate the spirituality subscale. **p* < .05, ***p* < .01, ****p* < .001.

As shown in Figure S9, spirituality moderated the mediating effects of risk perception by a significant interaction between perceived risk and spirituality on emotional distress (*β* = −0.17, 95% *CI* = [−0.27, −0.08], *p* = .004). Furthermore, the results of mediating effects of risk perception showed that the indirect effect of risk perception at high level (1 *SD* above the mean) of spirituality (*β* = −0.01, 95% *CI* = [−0.04, 0.03]) is weaker than that at low level (1 *SD* below the mean) of spirituality (*β* = 0.10, 95% *CI* = [−0.003, 0.20]), indicating that the mediating effects of risk perception between COVID-19 information exposure and emotional distress weakened with the increase of spirituality. To be specific, as indicated in Figure S10, at low levels of spirituality, greater perceived risk predicted severer emotional distress (*β =* 0.50, 95% *CI* = [0.26, 0.74], *p* = .000). However, at high levels of spirituality, the prediction of risk perception on emotional distress is not significant (*β* = −0.01, 95% *CI* = [−0.19, 0.16], *p* = .916).

*
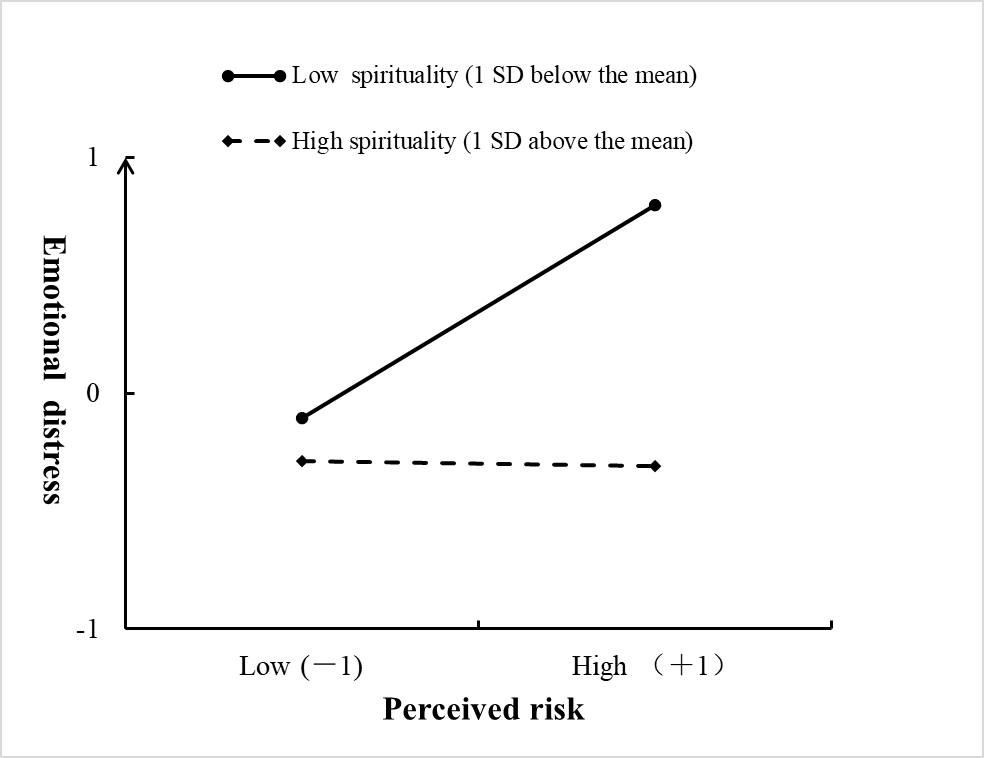
*

**Figure S10.** The simple slope analysis for the moderating effects of spirituality

*Note*. The dash line represents that the prediction of perceived risk on emotional distress is not significant.

**Reference**

Connor, K. M., & Davidson, J. R. (2003). Development of a new resilience scale: The Connor‐Davidson resilience scale (CD‐RISC). *Depression and anxiety*, *18*(2), 76-82.

Haddadi, P., & Besharat, M. A. (2010). Resilience, vulnerability and mental health. *Procedia-Social and Behavioral Sciences*, *5*, 639-642.
